# Effect of Dry–Wet Ratio on Properties of Concrete Under Sulfate Attack

**DOI:** 10.3390/ma12172755

**Published:** 2019-08-27

**Authors:** Jin-Jun Guo, Kun Wang, Ting Guo, Zheng-Yun Yang, Peng Zhang

**Affiliations:** School of Water Conservancy Science and Engineering, Zhengzhou University, Zhengzhou 450001, China

**Keywords:** concrete, sulfate erosion, dry–wetratio, microstructure

## Abstract

In order to explore the drying–wetting cycle test method of concrete under sulfate accelerating erosion, the influence of dry–wet time ratio on concrete sulfate erosion was studied. Under the condition of 7 days for one cycle, five different dry–wet time ratios were designed: 1:3, 1:1, 3:1, 5:1, and 10:1. The basic properties such as compressive strength, splitting tensile strength and dynamic elastic modulus of concrete were tested. Scanning electron microscopy (SEM) was used to analyze the microstructure of concrete before and after erosion. The test results show that under the environment of sulfate drying–wetting cycle erosion, the change of mechanical properties of concrete are divided into three stages: ascending period, fluctuating period and rapid descending period. Concrete is subjected to periodic damage process of initial damage followed by filling compaction, cracking, further filling, and cracking again, in that order. Dry–wet ratio has a significant effect on concrete sulfate attack. Under the same drying–wetting cycle period, as the dry–wet ratio increases, the degree of deterioration of concrete by sulfate attack increases first and then decreases. When the dry–wet ratio is 5:1, the deterioration is the most serious.

## 1. Introduction

Large amounts of concrete structures are being subjected to the erosion of different salt ions in their service environment [1,2,3]. Sulfate erosion is one of the main factors deteriorating the property of concrete. The soil in northwestern China and the coastal area is rich in SO_4_^2−^, which migrates into the concrete and undergoes a series of physical and chemical reactions to form ettringite and gypsum, and gradually stresses the inner wall of the pore, leading to plastic deformation and causing damage to the concrete structure [4,5]. The addition of class F fly ash can effectively improve sulfate resistance of concrete, while the strength of concrete will be reduced and chloride penetration of concrete will be increased at the same time [6]. The expansion of early age α-calcium sulfate hemihydrate (αHH) can decrease the shrinkage strain of cement mortar, and the generation of ettringite is more effective when αHH is substituted into Portland blast-furnace slag cement (PSC) than into ordinary Portland cement (OPC) and is thus more effective in suppressing the shrinkage strain [7]. In the drying–wetting alternate environments, such as water level change zones, ocean wave splash zones, and tidal zones, etc., the ions are transported into the interior of the concrete during the wetting process relying on internal and external humidity gradient deficit for concrete buildings (such as dams, port terminals, bridges, foundations, underground drainage pipes, etc.). The drying process enhances the internal ion concentration of the concrete by evaporation of water, and the volume expansion of the crystalline product can be up to 4 to 5 times [8], thereby enhancing the intensity of ion corrosion. According to related research, drying–wetting cycle environment is the most significant factor in the process of concrete erosion by sulfate, and the degradation rate is much higher than that of long-term immersed concrete [9]. As a consequence, studying the drying–wetting cycle system and its damage mechanism in concrete sulfate erosion process is the key scientific issue in water conservancy project, civil engineering, municipal engineering and traffic engineering. It can not only explore how to reasonably simulate the drying–wetting alternate process in the actual environment, promote the establishment of a unified drying–wetting cycle system standard in this field, but also benefit the formulation of indoor concrete drying–wetting cycle test procedures and systems. Improving the experimental research system of sulfate-eroded concrete has important engineering significance for improving the durability of concrete in harsh environments.

At present, there are many studies on the erosion performance of concrete under drying–wetting cycling conditions [10,11,12,13,14,15]. However, the dry and wet circulation methods adopted by various researchers are different without auniform theoretical basis, and there is no standard to follow. For instance, Jaya et al. used drying at 28 ± 3 °C for 9 h, wetting for 15 h, and simulating the tidal environment of the Straits of Malacca at a cycle of 24 h [10]. Garzón-Roca et al. studied the effect of drying–wetting cycle on the bond performance of concrete by immersing in solution at 20 ± 1 °C for 12 h and drying at 30 °C for 12 h [11]. Tang et al. selected immersion in salt solution for 16 h, drying at 80 ± 5 °C for 6 h, and cooling in a drying oven for 2 h, to study the resistance of concrete with coral reef sand to sulfate attack. Some scholars have begun to make a preliminary exploration for the wet and dry cycle system [12]. Sutrisno et al. controlled the test temperature at 30 °C and studied the effect of two dry–wet ratios (7:1 and 5:3) on the chloride content in concrete using an 8 h cycle [13]. Sobhan et al. used a dry-to-wet ratio of 1:1 and cycle periods of 12 h, 24 h, and 48 h to simulate the coastal environment in Iran and other regions [14]. Xu et al. studied two kinds of dry and wet cycle (48 h, 72 h) and five dry–wet ratios (1:1, 3:1, 5:1, 1:5 and 1:3) on the effect of concrete chloride ion transmission efficiency, and their results show that the ion diffusion efficiency decreases with the increase of dry–wet cycle and increases with the increase of dry–wet ratio [15]. The dry and wet circulation methods used by different researchers are different. There are three basic ideas: one is to simulate the engineering environment; the other is to make the concrete erosion speed the fastest; the third is to facilitate the test operation. This leads to poor comparability between research results from different researchers. Although some researchers have studied the performance of eroded concrete under different dry–wet ratios, they mainly focus on the transport of chloride ions. Chloride ions bring about structural deterioration caused by the corrosion of steel in concrete; the corrosion mechanism of chloride ions on concrete is different from sulfate ions [16,17,18]. Therefore, it is necessary to carry out experimental research on the degradation performance of concrete sulfate erosion under different dry–wet ratios, and to clarify the deterioration mechanism of dry–wet ratio to concrete, so as to formulate a reasonable dry–wet cycle system.

Compared with the mechanism of concrete sulfate degradation, the coupling mechanism of sulfate and drying-wetting erosion is not clear and is more complicated, so there is no consensus yet. Many researchers have carried out a lot of research on the macroscopic mechanical properties of concrete after sulfate attack under the self-designed drying–wetting cycle mode. Strength is used as an indicator to evaluate the deterioration of the performance of sulfate-eroded concrete under dry and wet cycles, such as compressive strength and splitting tensile strength. Ganjian et al. found that SF cement (silicon powder content 7% and 10%) has a lower compressive strength loss rate than ordinary concrete, and has better resistance to dry and wet cycles [19]. He et al. carried out a systematic experimental research, and the results showed that the compressive strength of the whole immersion specimen increased slowly, and the compressive strength of the drying and wetting cycle specimen suddenly dropped after a long time of microliter exposure [20]. Gao et al. used compressive strength and splitting tensile strength as indicators to study the deterioration of sulfate attack under the drying–wetting cycles of concrete with different mix proportion [21]. The results show that splitting tensile strength is more sensitive to damage compared with compressive strength. In addition, dynamic elastic modulus and mass loss are also often used to characterize the degree of deterioration of sulfate-eroded concrete. For example, Wang Qin et al. compared the dry–wet cycle with the full-immersion test piece [22]. The results show that the dry–wet cycle accelerates the rate of sulfate attack, resulting in a slight increase in the dynamic elastic modulus at the initial stage of erosion, and a sharp decline in the later stage of erosion. Gao et al. studied the effects of concrete sulfate attack coupling in dry–wet cycle on durability under dynamic load modulus and mass loss rate [23]. The results show that the dry–wet cycle reduces the resistance of concrete to sulfate attack, and the dynamic elastic modulus is more sensitive to concrete degradation than mass. The dry and wet circulation methods used by different researchers are diverse, lack the same theoretical basis, and there is no standard to follow. The dry–wet ratio, the dry–wet cycle, and the drying–wetting method are different. There are many differences in the conclusions drawn, and the comparability between the research results is poor. The wet and dry cycle is only a deterioration condition, and the different dry–wet ratios are not studied for the research object, revealing its influence on the long-term performance of concrete. So far, the unified wet and dry cycle system standards have not yet reached a consensus, which restricts the focus and development of concrete sulfate erosion research.

Considering the diurnal and semi-diurnal tides, the average infiltration time of the water level in different elevations, and the hydrometeorological data of the saline area in northwest China [24,25], five different dry–wet ratios (1:3, 1:1, 3:1, 5:1, 10:1) were selected to conduct the concrete sulfate attack experiment in the drying–wetting cycle environment. By studying the effect of dry–wet ratio on the strength, dynamic elastic modulus and mass change of concrete, and analyzing the evolution of concrete microstructure, the mechanism of concrete sulfate erosion is explored. The most unfavorable dry–wet time ratio is proposed in order to provide a theoretical basis for the development of relevant test procedures for concrete under sulfate attack environment.

## 2. Experimental Program

### 2.1. Materials

A Chinese standard ordinary Portland cement (OPC, similar to ASTM C150 Type I cement) of PO42.5R produced by a local manufacturer was adopted. Grade I fly ash was from the thermal power plant. Fine aggregate was natural river sand with the maximum size of 4.75 mm, fineness modulus (F.M) of 2.87 and absorption of 1.05%. Coarse aggregate of basalt with a diameter of 5–20 mm was used in this study. The common tap water was used as mixing water. The chemical composition of cement and fly ash is shown in Table 1. The physical properties of the cement are shown in Table 2. The water–binder ratio (W/B) adopted in this study was 0.54, and the fly ash was added by replacing the same amount of cement. The content of fly ash in this study was 20% and this content is often used in actual projects in China. Studies have shown that replacing cement with 20% by mass of fly ash can effectively improve the resistance of concrete to sulfate attack [26]. Sodium sulfate was purchased from a company in Shandong province, and the particle size was 120 meshes. The mixture proportion and corresponding compressive strength of the concrete are presented in Table 3.

### 2.2. Specimen Preparation and Curing Conditions

Concrete specimens were prepared in 60 liters compulsory mixer, and all the specimens were prepared from the same batch of concrete with three specimens per set. Cement, fly ash, sand and coarse aggregate were added to the mixer, stirred for one minute, then added with water, and finally mixed and stirred for 2 min. Immediately after mixing, the fresh concrete mixture was placed in molds to prepare the test specimens. After pouring, the molds with fresh concrete composite were placed on a concrete vibrating table for one minute for compaction. The samples were demolded after 24 h and then placed in a standard moist room (T = 20 ± 2 °C and RH > 95%) to be cured for 28 days.

### 2.3. Drying–Wetting Cyclic System

**Figure 1:**
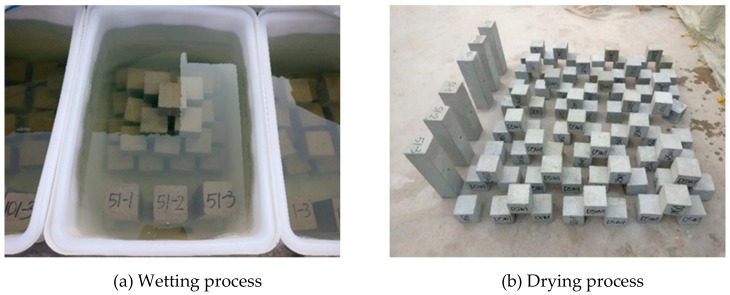
Concrete specimens exposed to sulfate solutions under drying–wetting cycles. (**a**) Wetting process; (**b**) drying process.

Referring to the standards of ASTM C1012-18a [27], ASTM C452-15 [28], GB/T 50082-2009 [29] and CECS 207-2006 [30], 5% Na_2_SO_4_ solution was selected as the corrosion solutions in this study. Five different dry–wet ratios (1:3, 1:1, 3:1, 5:1 and 10:1) were designed in the test, as shown in Table 4. Group W was used as a control group and was immersed in the same concentration sodium sulfate solution from the beginning to the end. In the dry–wet cycle test (Figure 1), the specimen was first immersed in a sodium sulfate solution (laboratory environment 25 ± 5 °C), with a corrosion-resistant polyethylene water tank selected as the solution container. When the immersion time reached the design time, the specimen was taken out and placed on the outdoor floor to dry naturally. In order to prevent variation in the concentration of the solution after too long of a time, it was necessary to periodically check and replace the solution to keep the concentration constant.

### 2.4. Experimen Methods

The test was carried out for 36 drying–wetting cycles during the course of 252 days. The strength, mass, and dynamic elastic modulus of the concrete specimens were measured every 28 days. In order to completely and uniformly etch all the samples, the volume ratio of the solution to all the samples was maintained at about 4:1, and the spacing of each sample was not less than 5 mm, furthermore, it was necessary to ensure that the liquid level exceeded 50 mm of the concrete sample.

Three specimens with the size of 100 mm × 100 mm × 400 mm were weighed with an electronic balance (resolution of 0.1 g), and the mass of each specimen was recorded once every 28 days’ exposure. The mass change (*M_c_*) of each group can be calculated from the average of three samples by Equation (1).

(1) $$M_{c}=\frac{m_{n}-m_{0}}{m_{0}}\times 100\%$$

where *m*_0_ and *m_n_* are the mass before and after *n* times of drying wetting cycles.

**Figure 2:**
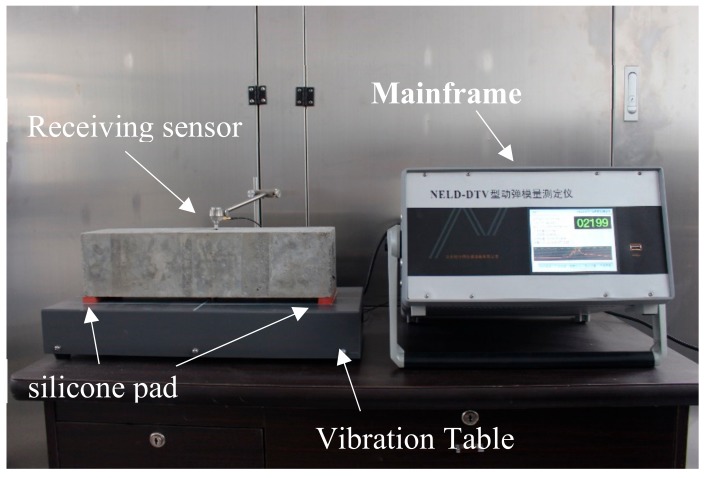
Dynamic elastic modulus tester.

The measurement of dynamic elastic modulus was conducted according to Chinese standard GB/T 50082-2009 [29]. The instrument used in this test is a dynamic modulus tester, which is shown in Figure 2.

The side perpendicular to the specimen molding surface was selected as the test surface and the middle point was marked as the measurement point, which avoided visible holes or cracks. The frequency range was selected from 1000 to 3000 Hz. Each specimen should be tested more than twice, and the frequency measurement error of the tests should not exceed 5 Hz. Then the average value of the measured values of the dynamic elastic modulus was taken as the final measurement value. Three specimens per group were taken as the final value of the dynamic elastic modulus of the concrete. The dynamic elastic modulus is calculated by Equation (2).

(2) $$E_{d}=13.244\times 10^{-4}\times mL^{3}f^{2}/a^{4}$$

where *m* (kg) is the mass of specimen, *L* (mm) is the total length, *f* (Hz) is the fundamental vibration frequency, *a* (mm) is the length of cross section. The unit for *E_d_* is MPa.

The Relative dynamic elastic modulus (RDEM (*E_rd_*)) can be calculated by Equation (3).

(3) $$E_{rd}=\frac{E_{t}-E_{0}}{E_{0}}\times 100\%$$

where *E*_0_ and *E_t_* are the dynamic elastic modulus before and after *t* times of drying–wetting cycles.

The time for mass measuring was consistent with the time for dynamic elastic modulus measuring, and the measurements should be carried out at the end of this drying process to prevent the influence of moisture content on the experiment. The test of strength was conducted according to Chinese standard GB 50081-2002 [31]. Compressive strength and splitting tensile strength tests were conducted on an Electro-Hydraulic Servo universal testing machine with a load capacity of 1000 kN, and the loads were applied at a rate of 0.5 MPa/s and 0.05 MPa/s respectively. The peak load was recorded during the test by an acquisition system.

The compressive strength, *f_cc_* (MPa), was calculated by Equation (4).

(4) $$f_{cc}=\frac{P}{A}$$

where *P* (N) is the peak load, *A* (mm^2^) is the pressure area of the specimen. The splitting tensile strength, *f_ts_* (MPa), was calculated using Equation (5).

(5) $$f_{ts}=\frac{2P}{\pi A}=0.637\frac{P}{A}$$

where *P* (N) is the peak load, *A* (mm^2^) is the fracture surface area of the specimen. The compressive strength and splitting tensile strength change of corroded concrete specimens could be characterized using the relative compressive strength coefficient *R_cc_* and the relative tensile strength coefficient *R_cs_*, which could be calculated by Equations (6) and (7).

(6) $$R_{cc}=\frac{f_{cc}{}^{n}}{f_{cc}{}^{0}}$$

(7) $$R_{cs}=\frac{f_{st}{}^{n}}{f_{st}{}^{0}}$$

where *f_cc_*^0^ and *f_cc_^n^* are the compressive strength before and after *n* times of drying wetting cycles; *f_st_*^0^ and *f_st_^n^* are the compressive strength before and after *n* times of drying wetting cycles.

The microstructure of the concrete was imaged with a scanning electronic microscope (SEM). Using the difference in gray scale of the image, erosive products such as ettringite, gypsum and thernadite could be distinguished. In order to explore the correspondence between microstructural changes and concrete macroscopic properties, a concrete core drilling machine was used to drill a core sample having a diameter of 20 mm and a height of 100 mm at the center of the bearing surface of the concrete specimen, and a thin film having a thickness of 2 mm was cut at a distance of 5 mm from the erosion surface of the cylindrical core sample by using a cutter. The sections were dried in a dry box for 12 h before the observation test. This test used platinum as the spray material, coating thickness was 5–20 nm and the test piece was attached to the copper production with conductive adhesive.

## 3. Results and Discussion

### 3.1. Visual Inspection

**Figure 3:**
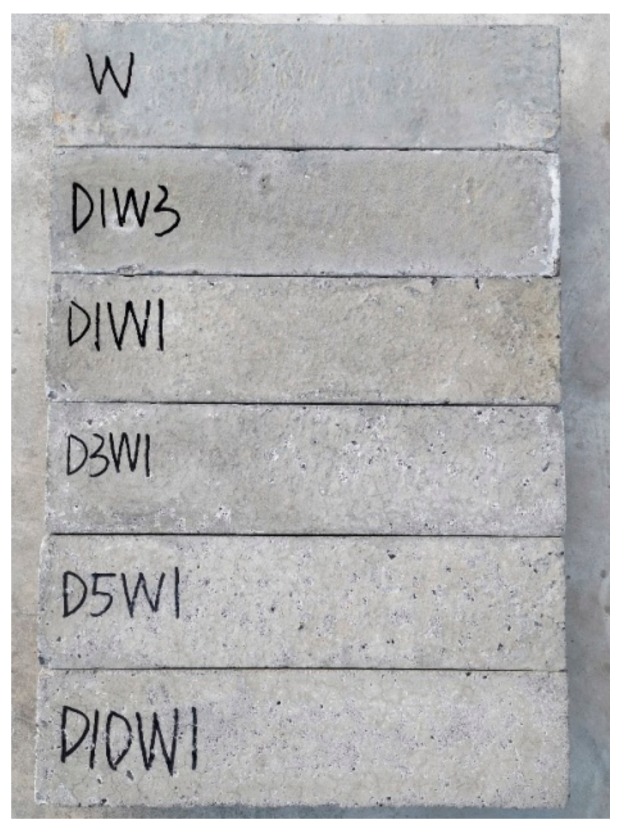
Visual inspection of concrete exposed to sulfate solution under drying–wetting cycles.

It is clearly observed from Figure 3 that the degree of surface deterioration is obviously different under five types of dry–wet ratios after 252 days. The degree of concrete surface damage increases with the increase of dry–wet ratio.

**Figure 4:**
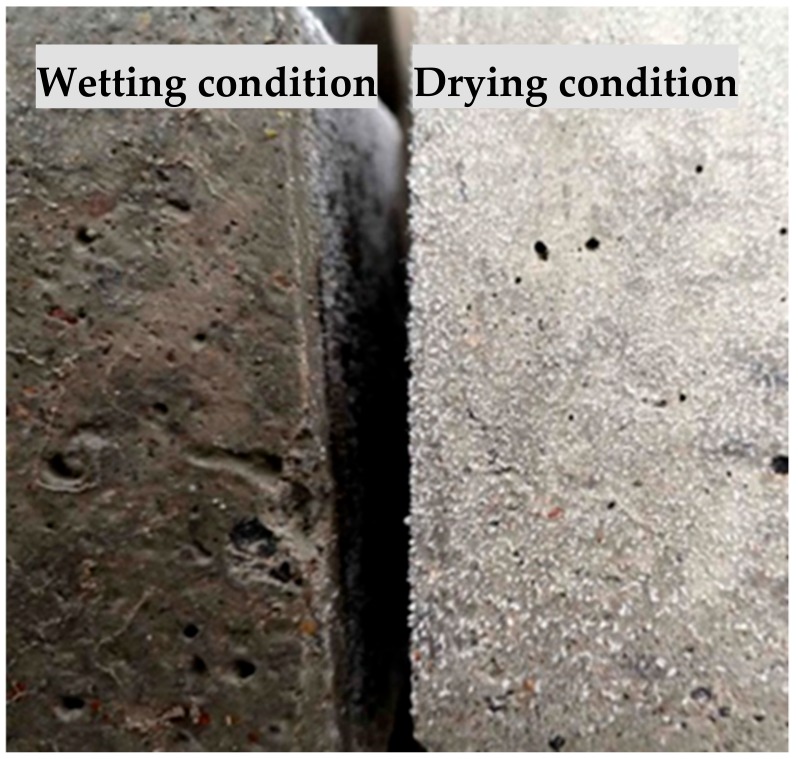
Comparison of salt crystal distribution on concrete surface between wetting and drying conditions.

As is shown in Figure 4, no sodium sulfate crystals are precipitated in the wetting state. In the drying state, the evaporation of water resulted in continuous concentration of salt solutionfrom the surface to the inside of concrete. Once the supersaturated solution is formed, the white salt crystals are precipitated and grow rapidly. In this process, a supersaturated film layer is formed between the crystal and the pore wall, and there is an energy difference between the crystal and the pore wall, which allows the crystal to continue to grow toward the pore wall, forming crystallization pressure, which will cause concrete cracking degradation [32,33,34].

### 3.2. Mass Change of the Concretes After Drying–Wetting Cycles

**Figure 5:**
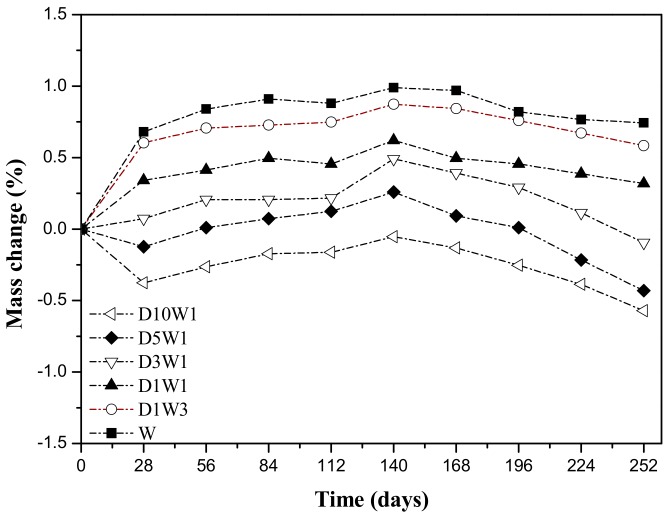
Mass change of specimens exposed to sulfate solution under different drying–wetting cycle systems.

As shown in Figure 5, the mass of the concrete changes is very little during the entire dry and wet cycle test, and the overall trend is firstly increased and then decreased. In the first 28 days, the mass of concrete with a dry–wet ratio of less than 5:1 increased, and the smaller the dry–wet ratio, the faster the mass grew. The mass of concrete with a dry–wet ratio greater than 5:1 rose slowly from 28 days after a short period of decline. After 140 days of dry and wet cycles, each group of specimens reached a peak and then began to decrease. The decrease rate in mass change of specimen increased with the increase of dry–wet ratio except for the D10W1 specimen. Themass of D5W1 concrete specimen has the largest decrease, with a 0.43% drop compare toinitial value and a 0.69% drop compared to peak value. There are several main factors affecting the quality change of concrete specimens. First, the sulfate ions react with the cement hydration to produce the swelling products such as ettringite, gypsum, and the mirabilite formed by the crystallization of the salt, filled the internal pores of concrete, thereby increasing the mass. Secondly, during the reaction, the dissolution and decomposition of components such as CH and C-S-H cause the concrete to be damaged and the quality is degraded. Thirdly, the peeling of the concrete by the erosion layer also causes the quality to drop rapidly. In the initial stage of erosion, the first influencing factor plays a leading role, that is, the erosion product fills the concrete pores and the concrete quality increases rapidly; as the erosion continues, the second and third influencing factors play a predominance function, and the mass of specimen begins to decrease.

### 3.3. RDEM of the Concretes after Drying–Wetting Cycles

**Figure 6:**
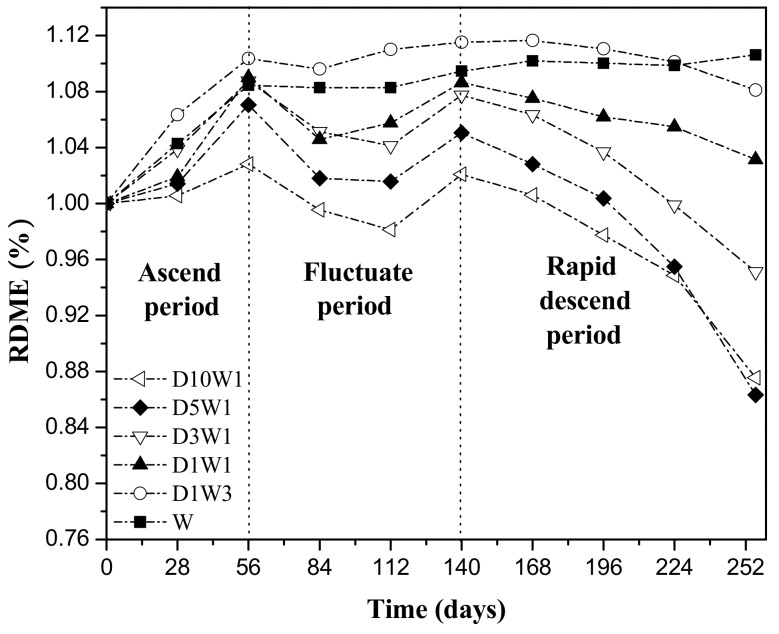
Change of RDEM of specimens exposed to sulfate solution under different drying–wetting cycle systems.

It can be seen from Figure 6 that during the whole erosion stage the RDEM change of concrete under dry–wet cycles are similar, and the whole is divided into three stages: ascending period, fluctuating period and rapid descending period. However, the RDEM of concrete that had been immersed the entire time generally showed a continuous upward trend, after being degraded by sodium sulfate for 252 days, the RDEM increased by 10.6% compared with the initial condition. During the ascending period of dynamic elasticity modulus, the smaller the dry–wet ratio is, the greater the mass change increases. The RDEM peak of D1W3 specimen increased by 10.3% relative to the initial value, more than 3 times that of D10W1 specimen. One reason is that, in the initial stage, concrete hydration continues, and the immersion process is beneficial to the increase of RDEM. Another reason is that sulfate ions in the solution enter the concrete and react with CH and cement hydration products to form swelling products such as gypsum and ettringite, which fills the internal pores of the concrete and increase the compactness.

During the fluctuating period, the RDEM of dry–wet cycle specimen re-raised to a peak after experiencing a period of decline. This is because the micro-regions inside the concrete cannot withstand the pressure generated by crystal crystallization, resulting in the creation of new micro-cracks, which in turn provide new growth space for the expansive products. In fact, the concrete experienced a cyclical damage process of "steady state—unsteady state—new steady state" at this stage. In addition, the silicon oxide and alumina in fly ash can generate secondary hydration reactions to form C-S-H and C-A-H under the excitation of cement hydration product Ca(OH)_2_ perhaps due to the active effect of fly ash, which causes an increase in dynamic modulus [35,36].

After 140 days, the concrete entered a rapidly descending period. The RDEM of D5W1 specimen has the fastest decline rate, which is 13.7% lower than the initial value at 252 days, while the W specimen is still slowly increasing. Drying has a more serious effect on the deterioration of concretein the later stage of sulfate dry–wet cycle erosion. The reason is that, during the drying process, as the relative humidity inside the concrete gradually decreases, the solution is easily over-saturated. Under the action of supersaturation, the solute particles move toward the nucleus and form an orderly accumulation on the surface, causing the nucleus to grow and form crystals. The crystal grows in a limited space and produces a huge crystallization pressure, which can damage the concrete [37]. In the wetting stage, the sulfate enters into the concrete through capillary action or diffusion, and chemically reacts with the cement hydration product to produce the swelling material. On the one hand, the increase in the wetting time can cause the micro-void and microcracks inside the concrete to be filled earlier by the expanded product. On the other hand, the increase in drying time can cause the crystal growth to generate a larger crystallization pressure. The interaction between the two factors ultimately leads to deterioration of the concrete.

### 3.4. Strength Change of Concrete under Drying–Wetting Cycles

**Figure 7:**
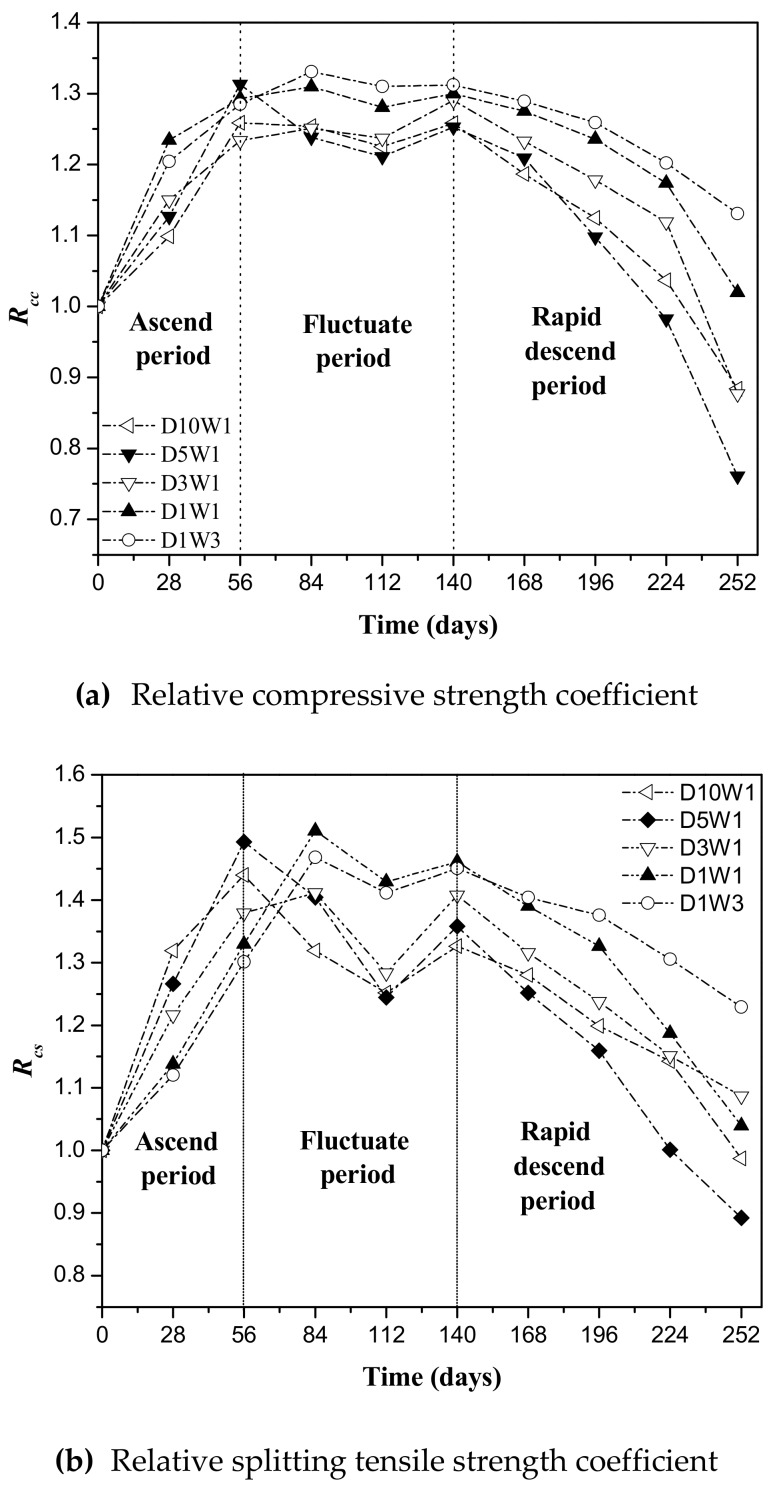
Mechanical properties of concrete exposed to sulfate solution under different dry–wet ratio. (**a**) Relative compressive strength coefficient; (**b**) relative splitting tensile strength coefficient.

Figure 7 shows the change trend of concrete compressive strength and tensile strength. It can be seen that the relative compressive strength coefficient and the relative splitting tensile strength coefficient of eroded concrete are basically consistent with the trend of RDEM, and are also divided into ascend period, fluctuate period and rapid descend period. During the ascending period, the mechanical properties of concrete have been greatly improved. The performance of concrete with larger dry–wet ratio is improved faster. The mechanical strength of D10W1 and D5W1 concrete first reaches the peak at 56 days, and the other three groups which have relatively small dry–wet ratiosalso reach a peak at 84 days. This is because the drying for a long time directly affects the migration of moisture in the concrete, resulting in low concrete saturation. Therefore, in the subsequent wetting process, the transport speed of the solution in the concrete is relatively faster, affecting the depth of the concrete is relatively deeper, resulting in higher transmission efficiency, and more favorable for the formation of the expansive products gypsum and ettringite.

As with RDEM, after experiencing a period of fluctuation, the mechanical properties of concrete fell rapidly. The rate of deterioration increased first and then decreased as the dry–wet ratio increased. The mechanical properties of D5W1 concrete decreased the fastest, and the compressive strength and splitting tensile strength of 252 days decreased by 24% and 11%, respectively, relative to the initial value. This finding can be explained by that, at the primal phase, a large amount of corrosion products accumulate in the pores inside the concrete to make the concrete dense, but excessive corrosion products cause macroscopic cracks in the specimen, and the concrete crack further expands and connects, becoming a fast passage for aggressive ion transport, which leads to an accelerated deterioration of the mechanical strength of the concrete [38,39]. Moreover, the interface between the slurry and the aggregate forms interfacial transition zone (ITZ) with higher porosity and pore size. Here, it is usually a vulnerable spot of concrete [40]. The expansion product produced by ITZ has more serious damage to the performance of concrete. Therefore, the reduction in ITZ strength is also one of the reasons for the deterioration of the mechanical properties of concrete [41,42]. This reduction in ITZ strength was also found in cement paste with recycled coarse aggregates (RCA) [43]. Compared with the compressive strength, the concrete splitting tensile strength is more sensitive to deterioration, which can better reflect the performance evolution of sulfate-eroded concrete. The dry–wet ratio plays an important role in the internal characteristics of the concrete. The faster the dry–wet ratio of the concrete improves the performance in the early stage of erosion, the faster the deterioration of concrete in the later stage of erosion.

### 3.5. Microstructural Investigations by SEM

**Figure 8:**
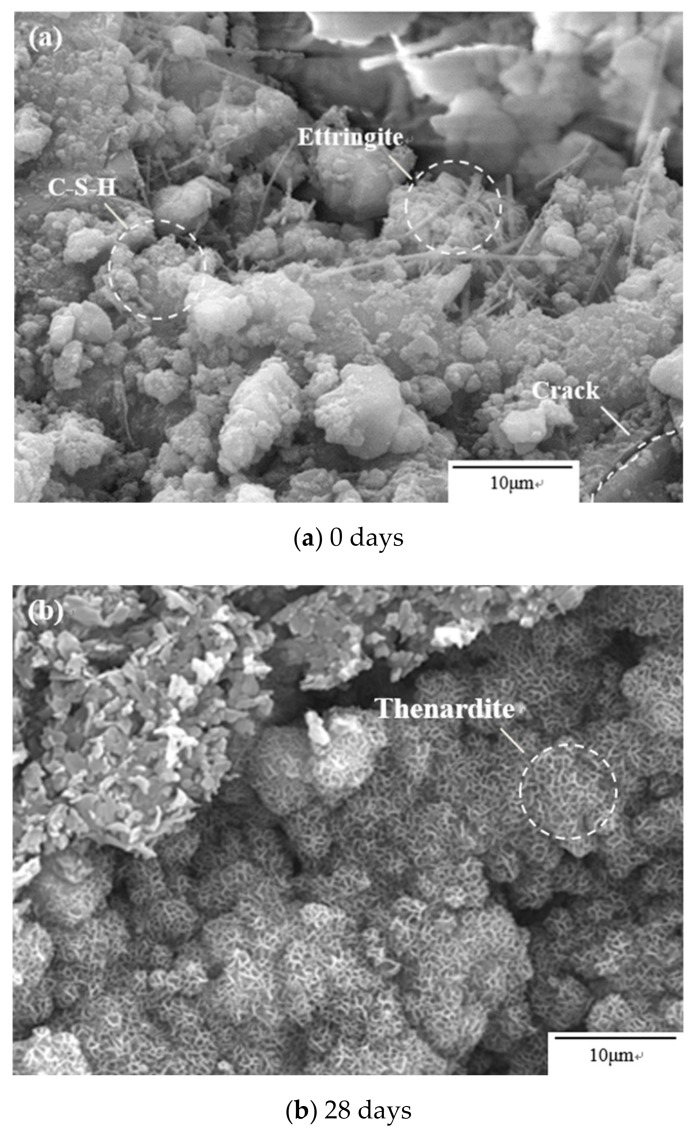

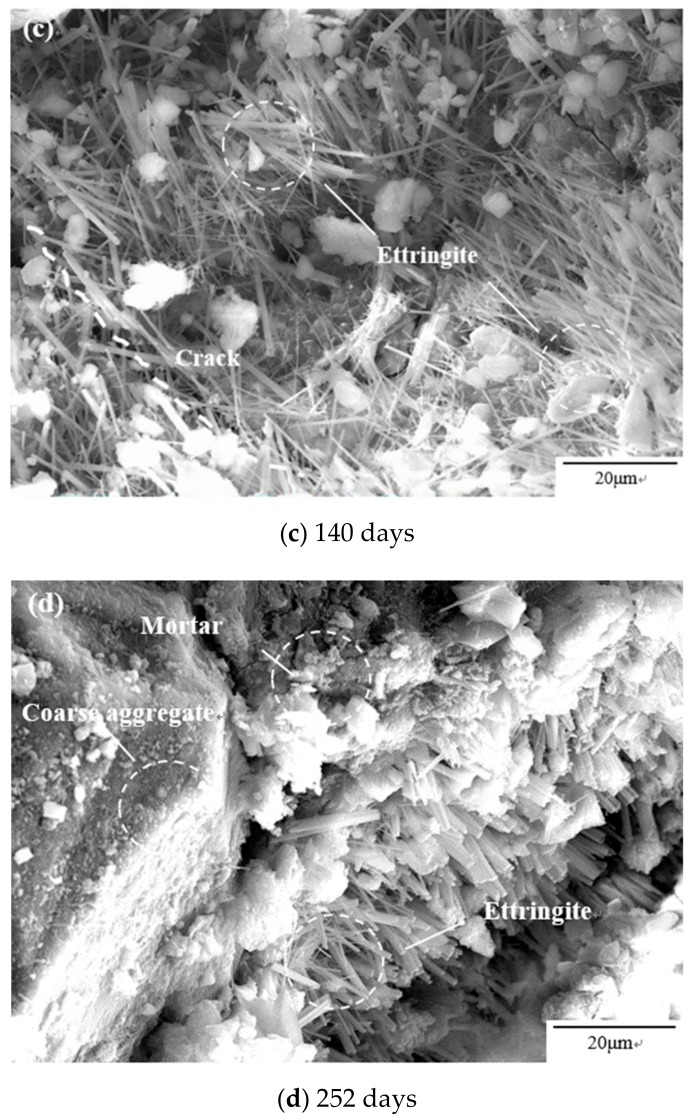
Microstructure of sample D5W1 exposed to sulfate solution under drying–wetting cycles. (**a**) 0 d; (**b**) 28 d; (**c**) 140 d; (**d**) 252 d.

Figure 8 is a microscopic picture of D5W1 concrete subjected to sulfate attack at 0, 28, 140, and 252 days under dry and wet cycles. The evolution of concrete performance was further analyzed by understanding the relationship between microstructure and strength, dynamic elastic modulus of concrete at different corrosion ages. Due to the hydration in cement, a small amount of needle-like ettringite crystals with a small volume and a large amount of fibrous material C-S-H can be observed in uneroded concrete as is shown in Figure 8a, and obviously, unspoiled concrete has high compactness. In spite of this, the dry shrinkage of the concrete still causes the initial damage of a small amount of irregular microcracks and micropores in the internal cement slurry.

Figure 8b shows a large amount of physically corroded products, which are thenardite crystals, are attached to the concrete surface in a form of net structure after 28 days of drying and wetting cycles. At room temperature, the product of sodium sulphate crystallization is mainly mirabilite. With the decrease of relative humidity, mirabilite (Na_2_SO_4_·10H_2_O) gradually becomes unstable and begins to change to thenardite (Na_2_SO_4_). The thenardite has greater crystallization pressure and produces more serious damage. In many real structures, most concrete members display contamination of soluble salts [44]. It can be concluded that the deterioration of the concrete during the drying process is achieved by changing the ambient humidity. During the course of transition from a high-humidity environment to a low-humidity environment, the changes in sodium sulfate crystal form generated enormous crystallization pressure, which means that physical erosion dominates the process.

As can be seen from Figure 8c,d, the ettringite crystals are distributed from the inside to the outside in a chaotic manner, and the crystal length is about 10–20 μm. With the increase of erosion age, the amount of erosion products increases as well. It is observed that the ettringite clusters are distributed, and the gypsum crystals appear in the form of slabs, and gathered together to form a radial distribution. The products of chemical attack of sulphate, ettringite, gypsum, etc., are mainly found in the micropores, micro-cracks, interfacial transition zones and structural damage zones of concrete. These areas provide plenty of growth space for ettringite and gypsum, partly due to concrete itself, the initial defect caused, and anotherpart of the new transmission passage formed by the original crack further expanding and connecting. Investigations have shown that halite, thenardite, hexaedrite, and carnaliteare found inside historical sites due to the erosion of soluble salts, which is one of the reasons for the real structural deterioration [44]. Chemical corrosion products (ettringite and gypsum) and physical erosion products (mirabilite and thenardite) fill the internal space of the mortar, which densifies the structure of the concrete, and the macroscopic performance is the improvement of mechanical properties. As the corrosion product increases, the expansion stress generated further increases. When it is larger than the flexural strength of the mortar, the structure is destroyed, and new microcracks are generated, and the macroscopic performance indicates a decrease in mechanical properties. The new crack continues to be filled with corrosive products, so fluctuations in concrete strength occur.

## 4. Conclusions

In this study, an experimental research on concrete exposed to sulfate solution under different dry–wet ratios was conducted. The mass, dynamic elastic modulus, compressive strength, and splitting tensile strength of concrete were measured, and SEM images of different corrosionages are also recorded. The conclusions are summarized as follows:

(1) Under sulfate erosion environments of different dry–wet ratios, the change of mechanical properties of concrete is divided into three periods: ascend period, fluctuate period, and rapid descend period. Under the same dry and wet cycle, as the dry–wet ratio increases, the degree of concrete deterioration increases first and then decreases. When the dry–wet ratio is 5:1, the degree of concrete deterioration is the most serious;

(2) The deterioration of concrete during the drying process is a physical reaction process, and the crystallization pressure caused by crystal crystallization and crystal transformation is an important cause of deterioration. The extension of drying time has a greater impact on the later stage of erosion;

(3) During the sulfate erosion process, the interior of the concrete undergoes aperiodic damage process of “initial damage—filling compaction—cracking—further filling—cracking again”. The concrete is dense to loose, and the concrete performance of RDEM and strength is initially rising, however after the fluctuating period it drops rapidly;

(4) Mass change can better characterize the deterioration of concrete, but the correlation with the change of mechanical properties is weak. The splitting tensile strength is more sensitive than the compressive strength.

## Figures and Tables

**Table 1:** Chemical composition of cement and fly ash.

Constituent (wt%)	SiO_2_	Al_2_O_3_	CaO	MgO	SO_3_	Fe_2_O_3_	Na_2_O	K_2_O	LOI
Cement	31.43	12.43	41.28	3.34	3.22	3.34	0.43	0.80	1.09
Fly ash	58	30	2.8	1.5	1.22	4.3	0.00	1.36	0.82

**Table 2:** Physical properties of the cement.

Standard Test Method for Water (%)	Stability (Boiling Method)	Setting Time (min)		Compressive Strength (MPa)		Flexural Strength (MPa)	
		Initial Setting	Final Setting	3 d	28 d	3 d	28 d
26.74	qualified	90	300	26.6	54.5	5.42	8.74

**Table 3:** Mix proportion and compressive strength of concrete.

Water–Binderratio	Cement/ (kg/m^3^)	Fly Ash/ (kg/m^3^)	Water/ (kg/m^3^)	Aggregate (kg/m^3^)	Sand/ (kg/m^3^)	Compressive Strength/(MPa)
0.54	289	72	195	1178	722	35.5

**Table 4:** Drying–wetting cycles test arrangements.

Code	D1W3	D1W1	D3W1	D5W1	D10W1	W
Ratio of dry and wet	1:3	1:1	3:1	5:1	10:1	0:1
Single time ratio/h	42:126	84:84	126:42	140:28	152:16	0:168

Note: D1W3 represents the specimen having a dry–wet time ratio of 1:3.
